# The role of heme oxygenase-1 in hematopoietic system and its microenvironment

**DOI:** 10.1007/s00018-021-03803-z

**Published:** 2021-03-31

**Authors:** Agata Szade, Krzysztof Szade, Mahdi Mahdi, Alicja Józkowicz

**Affiliations:** Department of Medical Biotechnology, Faculty of Biochemistry, Biophysics and Biotechnology, Jagiellonian University, Gronostajowa 7, 30-387 Krakow, Poland

**Keywords:** HO-1, Hmox1, HSPC, Hematopoiesis, Niche

## Abstract

Hematopoietic system transports all necessary nutrients to the whole organism and provides the immunological protection. Blood cells have high turnover, therefore, this system must be dynamically controlled and must have broad regeneration potential. In this review, we summarize how this complex system is regulated by the heme oxygenase-1 (HO-1)—an enzyme, which degrades heme to biliverdin, ferrous ion and carbon monoxide. First, we discuss how HO-1 influences hematopoietic stem cells (HSC) self-renewal, aging and differentiation. We also describe a critical role of HO-1 in endothelial cells and mesenchymal stromal cells that constitute the specialized bone marrow niche of HSC. We further discuss the molecular and cellular mechanisms by which HO-1 modulates innate and adaptive immune responses. Finally, we highlight how modulation of HO-1 activity regulates the mobilization of bone marrow hematopoietic cells to peripheral blood. We critically discuss the issue of metalloporphyrins, commonly used pharmacological modulators of HO-1 activity, and raise the issue of their important HO-1-independent activities.

## Introduction

### HSC and their niche, hematopoiesis

Hematopoietic stem cells (HSC) produce all the blood cells throughout life in a process called hematopoiesis. The main characteristics of HSC are self-renewal and differentiation toward all cells among the hematopoietic blood lineages (reviewed in [1, 2]).

The rare HSC sit at the apex of the hierarchical structure of the hematopoietic system. In 1988 Weissman laboratory isolated murine HSC and showed that this cell population differentiates to all mature blood lineages in the irradiated mice [3]. Further research revealed three distinct subpopulations at the top of hematopoietic tree: long-term HSC (LT-HSC), short-term HSC (ST-HSC), and multipotent progenitors (MPP) [4]. While all of these populations have multipotent differentiation potential, only the LT-HSC can self-renew over the entire lifetime, what is progressively lost as they differentiate toward ST-HSC and MPP. MPP differentiate downstream into two types of oligopotent progenitors: common myeloid progenitors (CMP) and common lymphoid progenitors (CLP), where myeloid oligopotent progenitors will give rise to bipotent megakaryocyte-erythrocyte progenitors (MEP) and granulocyte–macrophage progenitors (GMP) [5, 6]. Those oligopotent progenitors will eventually differentiate to unipotent progenitors and ultimately will give rise to downstream diverse mature progenies [7]. Several studies based on single-cell transplantation assays questioned, however, the classic hematopoietic hierarchical differentiation tree and suggested that subpopulation of HSC may directly differentiate to lineage-biased progenitors, skipping the MPP stage [8, 9].

The proper function of HSC tightly depends on the bone marrow (BM) microenvironment named 'niche'. This specialized niche sustains the multipotency and self-renewal of HSC and prevents their exhaustion. Therefore, the niche is critical for maintaining the hematopoietic homeostasis and regulating the response of hematopoietic system in stress conditions [10].

The conceptualization of stem cell 'niche' as a specialized microenvironment within the BM referred to studies done a few decades earlier by Schofield (1978). He proposed that the niche regulates HSC properties of multipotency, self-renewal, and quiescence via specific signals [11]. Over the years this concept was consequently confirmed by numerous studies that demonstrated many cellular and non-cellular components of the HSC-niche. The niche complex milieu provides the HSC with the essential physical interaction and the molecular cues that are critical for the differentiation, maintenance, and localization of HSC.

HSC niche is composed of several cell types. First findings showed a crucial role of stromal cells, and identified clonal BM stromal cells which support the HSC self-renewal, and the maturation of both CMP and CLP [12]. Of different mesenchymal stromal cells (MSC), CXCL12-abundant reticular (CAR) cells are essential for maintaining the quiescence of HSC [13]. CAR phenotype highly overlaps with the later-described leptin receptor-expressing MSC (LepR +) [14]. While it was initially indicated that HSC niche locates near the endosteum [15, 16], further studies suggested that the majority of HSC reside in direct proximity of blood vessels rather than near bone surface [17–19].

The development of the transgenic mouse models and advanced microscopy techniques facilitated the identification of subsequent cellular elements of HSC niche as essential factors in regulating HSC function. These included hematopoietic cell types such as megakaryocytes [20], and non-hematopoietic cell types such as MSC, adipocytes and glial cells, as well as niche-derived growth factors and signaling molecules, along with their receptors [21, 22].

HSC progenies are also able to regulate HSC activities in a feedback loop. For instance, HSC quiescence can be regulated directly by megakaryocytes [20, 23–25]. Bruns et al. described HSC subpopulation associated with megakaryocytes, and reported that depletion of megakaryocytes may trigger HSC proliferation [23]. After exposure to a lethal dose of radiation, megakaryocytes drive the repair of HSC niche via osteolineage cell differentiation [24].

Despite the extensive studies on HSC niche, some questions are still unanswered and some mechanisms underlying the HSC-niche interaction within the BM and their function are incompletely understood.

The existence of clonal precursors for hematopoiesis was suggested already in 1960′s [26, 27] and since then HSC became one of the most intensively studied subjects of research. Nevertheless, there are still a lot of unanswered questions and controversies concerning their biology and differentiation and their interactions with their niche [28]. Among many factors that were shown to regulate this system was heme oxygenase-1 (HO-1).

### Heme oxygenase-1

**Fig. 1:**
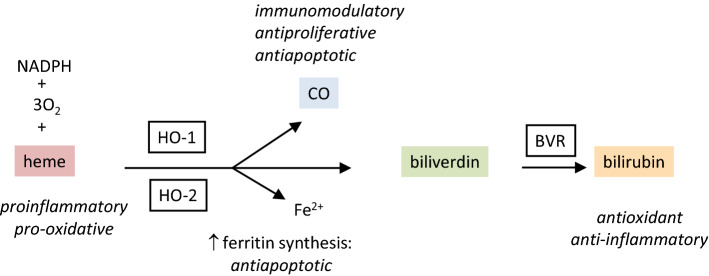
Catalytic reaction of heme oxygenase (HO-1 and HO-2). Heme is degraded into CO, Fe2^+^ and biliverdin. The reaction requires molecular oxygen and NADPH. Water-soluble biliverdin is further reduced to insoluble bilirubin by biliverdin reductase (BVR)

Heme oxygenase is an enzyme which degrades heme [29]. The products of the reaction catalyzed by heme oxygenase are equimolar amounts of carbon monoxide (CO), ferrous ions and biliverdin, which is subsequently converted to bilirubin by biliverdin reductase (BVR) [30]. Catalytic heme degradation requires an electron donor, that is NADPH provided by P450 cytochrome reductase and oxygen (reviewed in [31], (Fig. 1)).

There are two existing isoforms of heme oxygenase, HO-1 and HO-2 [32, 33], encoded by two different genes, HMOX1 and HMOX2 [31]. While HO-2 is a constitutive isoform, expressed mainly in the brain and testes [34, 35], HO-1 is induced by a variety of factors. The most obvious advantage of HO-1 activity is the removal of free heme, which is a known pro-oxidant, and a direct regulator of several transcription factors [36]. Moreover, heme can act as a damage-associated molecular pattern (DAMP) and activate innate immune response [37].

Of all the heme degradation products, CO seems to be the most important in the regulation of the immune system (reviewed in [31]). CO reduces the production of proinflammatory cytokines—IL (interleukin)-1, IL-6, TNFα (tumor necrosis factor α) and the expression of adhesion molecules, and simultaneously increases the production of anti-inflammatory IL-10 [38]. CO affects the transmission of signals from certain TLRs (Toll-like receptors), which are very important in the initiation of immune responses against pathogens [39]. CO can also act as an anti-apoptotic factor by modulating Fas/Fas ligand and Bcl-2 family [40].

The second of the heme degradation products, biliverdin, is almost instantly converted to bilirubin by BVR [41]. Bilirubin is a potent antioxidant and anti-inflammatory factor—it is an efficient scavenger of reactive oxygen species [42] and inhibits the adhesion molecules signaling [43, 44].

The last of the HO-1 reaction products, ferrous ions, can be considered harmful. However, the release of potentially pro-oxidant Fe^2+^ ions induces the expression of ferritin [45], which apart from sequestering iron, also can have an anti-apoptotic effects [46].

HO-1 might also directly regulate the cytokine expression [47]. Study by Ghoreschi et al. shows that fumarate treatment of DC upregulates HO-1, which is cleaved and translocated to the nucleus. In the nucleus HO-1 associates with AP-1 or NFκB binding sites in the IL-23p19 promoter, where it interferes with transcriptional activity. As a result, HO-1 decreases IL23p19 transcription [47].

A lot of data concerning the role of HO-1 in the normal and disease conditions were reported after the generation of genetically modified mice that lack the expression of HO-1 (HO-1^−/−^) [48]. HO-1^−/−^ mice are affected by a chronic proinflammatory state and dysregulated iron homeostasis [48].

HO-1 deficiency was also reported in humans, for the first time by Yachie et al. in 1999 [49]. Since then there were only a few other cases described [50–54]. However, it is possible that due to the unspecific and variable symptoms, such as fever, hemolytic anemia, hematuria, proteinuria, hypertension and growth retardation, many other patients are undiagnosed. All of the five described patients from India had the same homozygotic R44X mutation [53], what suggests, that this mutation exists in the population. Unfortunately, the majority of the affected children died due to pathological changes in various organs [50, 53, 54]. It was shown that the absence of macrophages in the spleen and liver and in consequence the inability to remove senescent erythrocytes (RBC) and hemoglobin from the circulation is the main cause of the disease in HO-1 deficient mice [55]. Based on experiments in mice, Kovtunovych et. al suggested, that bone marrow transplantation could work as a possible treatment of HO-1 deficiency in humans [56]. Indeed, one patient was successfully treated with hematopoietic stem cell transplant from his sister [57].

HO-1 deficiency luckily seems to be extremely rare, but there are significant differences in HO-1 expression/activity in the human population due to the HMOX1 promoter polymorphism [58, 59]. The differences in the number of GT repeats affect HO-1 expression: shorter alleles result in the higher basal expression and stronger induction by the HO-1 inducers than the longer alleles [60] and were linked to the risk of various diseases, such as diabetes [61], multiple sclerosis [62], chemotherapy-induced neutropenia [63] and cardiovascular diseases [64, 65].

## Role of HO-1 in HSC and progenitors

**Fig. 2:**
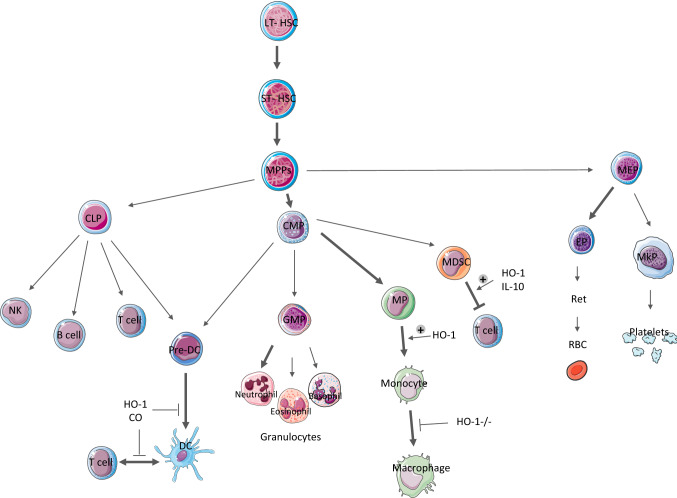
Simplified scheme of hematopoiesis and interactions of immune cells, showing the main processes which are regulated by HO-1 (bold lines), including the ones that are stimulated (arrows with „plus” symbol) and inhibited (bar-headed arrows) by indicated conditions

Due to the anti-inflammatory, antioxidative and anti-apoptotic properties of HO-1, it was hypothesized that HO-1 interferes with the HSC differentiation. The role of HO-1 in steady-state regulation of HSC is supported by our study based on HO-1 deficient mice (HO-1^−/−^) [66] (Fig. 2). The HO-1^−/−^ mice have increased numbers of strictly phenotypically defined LT-HSC, as well as MPP. Moreover, the LT-HSC from HO-1^−/−^ mice have a higher proliferation rate and show features of premature aging on global transcriptional and functional level [66]. This is connected with the increased DNA damage [66]. Although the altered biology of HSC in HO-1^−/−^ mice is evident, we suggest that it results at least in part, from the role of HO-1 in the BM niche (discussed in the next chapter).

Another study employed double mutant HO-1^−/−^Bach1^−/−^ mice [67]. In this model, the frequency of hematopoietic stem and progenitor cells (HSPC) is also increased, but the number of the common myeloid progenitors is decreased. This indicates that HO-1/Bach1 pathway is necessary for the commitment of multipotent progenitors to myeloid lineage [67]. However, the study did not analyze whether the same effect is observed in HO-1^−/−^Bach1^+/+^ model. Therefore, the direct role of HO-1 on the differentiation blockage at the CMP stage still has to be clarified, especially because HO-1^−/−^Bach1^+/+^ mice have higher numbers of circulating monocytes and granulocytes [66], what may seem inconsistent with reduced numbers of CMP in HO-1^−/−^Bach-1^−/−^ mice.

Bach1 and Bach2 are DNA-binding proteins, which work mainly as transcriptional repressors [68]. Heme regulates *Bach1* expression by binding to Bach1 protein and inducing its nuclear export [69]. Furthermore, heme was shown to induce the ubiquitination and proteasomal degradation of Bach1 [70]. Bach2 is repressing myeloid genes by direct binding to their promoters, thus promoting B cell development [71]. On the other hand, Bach1 and Bach2 redundantly repress *Hmox1* expression [72]. Heme binds to Bach1 and Bach2 and inhibits their activity, providing the negative feedback loop for the control of Hmox1 expression.

HO-1 is important for the proper function of HSC not only in steady-state conditions but also under stress. HO-1 expression in HSPC in steady-state levels is low, but increases in hematopoietic stress, such as myeloablation. Paradoxically, a study done on HO-1 heterozygous mice (HO-1^−/+^) showed that these mice recovered after the 5-FU-induced myeloablation faster than HO-1^+/+^ mice, which was linked to increased proliferation of HSPC [73]. However, despite the faster response to myeloablation, HSC in HO-1^−/+^ mice lost the potential to reconstitute irradiated recipients after serial transplantation [73]. Thus, it suggests that HO-1 regulates how HSPC respond to stress stimuli and protects them from exaggerated proliferation and premature exhaustion.

Heme is an important regulator of erythropoiesis, therefore HO-1, as a heme degrading enzyme, was also suggested to modulate erythropoiesis [74]. The early evidence pointing to the potential role of HO-1 in erythropoiesis was presented by Abraham et al. [75, 76]. While the initial report based on K562 cell line suggests that HO-1 level in erythroid cells might be negligible [77], further studies done on mouse model evidenced that HO-1 is expressed in erythroblasts and that its expression is increasing with erythroid differentiation [78]. It was also shown that both downregulation and upregulation of HO-1 dysregulates proper erythroid differentiation [78].

Importantly, HO-1^−/−^ mice present several disturbances in steady-state hematopoiesis and have microcytic anemia [79]. These erythroid defects may be caused not only by the lack of HO-1 in erythroid lineage but also by the lack of HO-1 in macrophages. The formation of erythroid cells occurs in so-called erythroblastic islands, that are composed by centrally located macrophage and adherent, differentiating erythroblasts [80]. The macrophages within the erythroblastic islands highly express HO-1, and HO-1-deficiency reduces their number and disturbs the formation of erythroblastic islands [79]. The crucial role of macrophages in erythroblastic islands may be connected with the enzymatic product of HO-1 reaction—carbon monoxide (CO) [79]. The in vitro model indicated that in low-oxygen conditions, that are typical for BM, CO prevented the death of erythroid precursors and triggered erythroid differentiation [81].

Finally, HO-1 plays an important role in stress hematopoiesis. In the transplantation model, which is used to simulate stress conditions, irradiated recipient mice that received HO-1^+/−^ cells had lower numbers of erythroblasts. This was linked with an increased level of TNFα in the splenic macrophages and decreased CD49d expression levels in proerythroblasts [82].

Upstream from HO-1, erythropoiesis is regulated by Bach1 and Bach2 [83, 84]. Bach1^−/−^Bach2^−/−^ mice have erythroblast-maturation disorder, which is connected with HO-1 de-repression. Moreover, downregulation of Bach1 and Bach2 during infection inhibits erythropoiesis, allowing for enhanced expression of myeloid genes and thus shifting the hematopoiesis towards the production of innate immunity cells [83, 84].

## Role of HO-1 in HSC niche

### Endothelial cells

HSC and endothelial cells (EC) are closely related from the very beginning of fetal development. Endothelial cells are one of the most crucial regulators of HSC niche in the bone marrow. At the same time, endothelial cells are one of the cell types, in which HO-1 plays a critical role. Since there are comprehensive reviews that cover the topic of the role of HO-1 in endothelial cells [64, 85–87], we only highlight the most important issues.

HO-1 protects endothelial cells from apoptosis [88], and this is dependent on the generation of CO [89]. In general, HO-1 can act as a pro- and anti-proliferative factor, depending on the cell type, tissue and health/disease status. However, in the case of endothelial cells, HO-1 increases their proliferation [60, 90]. Because of its influence on EC proliferation, but also due to the modulation of expression and activity of proangiogenic factors, such as SDF-1 and VEGF, HO-1 is an important regulator of angiogenesis (reviewed in [91]). Furthermore, HO-1 can inhibit inflammation by decreasing the expression of adhesion molecules (E-selectin and VCAM-1) on the surface of endothelial cells [92]. This occurs through the inhibition of NF-κB [93].

The importance of HO-1 for the proper function of endothelial cells is further evidenced by the human cases of HO-1 deficiency—the affected children suffered from severe endothelial cell injury due to oxidative stress [49].

### Mesenchymal stromal cells

Mesenchymal stromal cells (MSC) are also known by different names, such as multipotent stromal cells or—imprecisely—mesenchymal stem cells [94]. Although they can be found in different tissues, such as umbilical cord, adipose tissues, and placenta, the major source of MSC used in research is the bone marrow [95]. MSC can differentiate to osteoblasts, chondrocytes, and adipocytes [96].

MSC constitute an important part of the HSC niche. Moreover, human MSC transplanted subcutaneously form a humanized niche, able to support transplanted human HSC in mouse [97].

Several studies from Abraham group showed that HO-1 can also promote the MSC differentiation into osteoblasts and inhibit the MSC differentiation toward adipocytes while inhibiting HO-1 can promote adipogenesis [98–100]. However, other studies suggested that HO-1 overexpression has no effect on their differentiation potential [101, 102]. We and others have shown no differences in the differentiation potential between HO-1^+/+^ and HO-1^−/−^ MSC [103, 104].

Yu et al. reported the negative correlation between HO-1 expression level in the bone marrow transplantation (BMT) recipients and acute-graft-versus-host disease (aGVHD) associated with allogeneic HSC transplantation [105]. The authors further used HO-1-overexpresing MSC to modulate the Th17/Treg ratio and prevent GVHD in mice [105].

HO-1 was shown to be responsible for the immunosuppressive effect of rat MSC on T cells [106]. HO-1 inhibition abrogated the protective effect of MSC on the heart allograft rejection [106]. Additionally, the overexpression of HO-1 improved the regeneration potential of allogenic MSC in porcine model of myocardial infarction after intracoronary infusion [107].

**Fig. 3:**
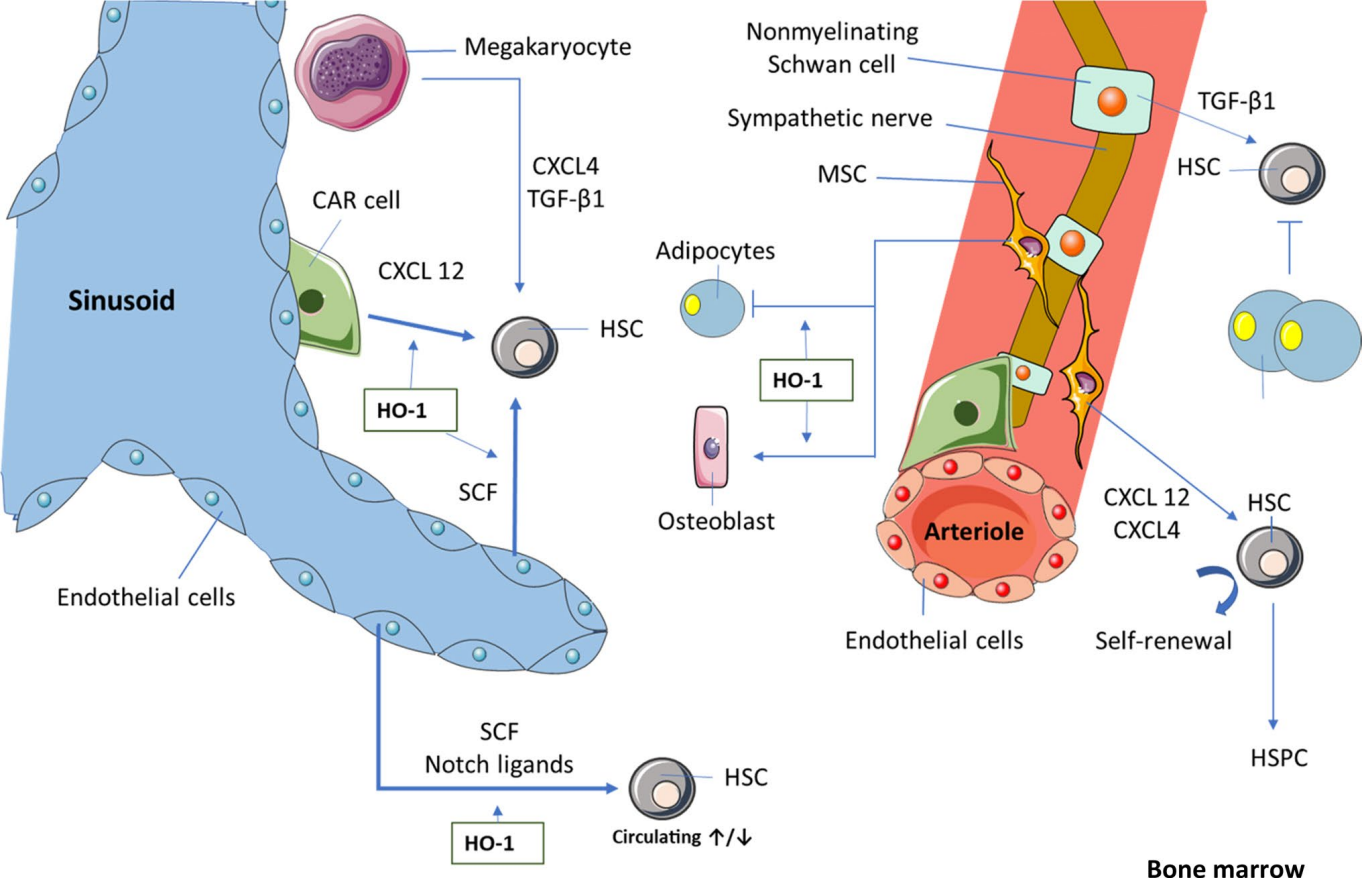
Influence of HO-1 on bone marrow HSC niche

Altogether, being an essential part of the BM niche, MSC can influence HSC and hematopoiesis, but also through their immunomodulatory activity, they can regulate the function of the mature immune cells. In both cases, HO-1 is an important player (Fig. 3).

BM endothelial and CAR cells constitute the most important part of the HSC niche [108]. We observed that these two cell types highly express HO-1 [66]. The frequencies of EC and CAR cells were altered in HO-1^−/−^ mice and they produced reduced amounts of TGF‐β1, SCF and SDF‐1α [66]—factors which are essential for maintaining the HSC quiescence [13, 109, 110]. Consequently, wild-type HSC transplanted into irradiated HO-1^−/−^ mice lost their hematopoietic potential, whereas HO-1^−/−^ HSC phenotype and functions were rescued when transplanted to the wild-type mice [66]. This suggests that the expression of HO-1 in HSC niche might be more important for sustaining the proper function of HSC than an expression of HO-1 in HSC themselves.

## HO-1 as a regulator of innate and adaptive immunity

As indicated above, HO-1 activity affects the levels of many inflammatory mediators, but the current knowledge points to a more complex role of HO-1 in mounting both the innate and adaptive immune response at the cellular level [111].

Participation of HO-1 in the regulation of innate immunity was mainly investigated in the context of monocyte/macrophage lineage function. These studies were triggered by initial observations that HO-1^−/−^ mice have elevated levels of MCP-1 (monocyte chemoattractant protein-1)—the proinflammatory factor regulating the response of monocytes and macrophages (reviewed in [112]). Further studies confirmed that lack of HO-1 causes the over-activated, proinflammatory phenotype of macrophages (reviewed in [111]). Otterbein et al. described the molecular mechanism that underlies this phenomenon. They showed that CO produced by HO-1 stimulates the production of IL-10 by macrophages [38]. Moreover, not only HO-1 activity stimulates the IL-10 production, but also IL-10 upregulates HO-1 expression [113]. This indicates the presence of a positive feedback mechanism and emphasizes the importance of HO-1 in anti-inflammatory function of IL-10.

The role of HO-1 in monocyte/macrophage lineage is not limited to the regulation of the function of mature macrophages. Recent study evidenced that HO-1 is involved in the maturation of the myeloid cells from hematopoietic stem and progenitor cells. The specific deletion of HO-1 in myeloid lineage (LysM^Cre/+^Hmox1^fl/fl^) reduced the differentiation of myeloid progenitors toward macrophages [114]. It was shown that CO, produced by HO-1, stimulates the differentiation of myeloid progenitors to macrophages, increases CD14 on their surface and enhances sensitivity to M-CSF (macrophage colony-stimulating factor) stimulation [114].

The importance of HO-1 already in early differentiation steps of myeloid development was further confirmed by its role in myeloid-derived suppressor cells (MDSC)—a population representing the immature myeloid cells circulating in the peripheral blood [115]. HO-1 was crucial for the immunosuppressive function of MDSC population in the IL-10-dependent manner [116].

HO-1 deficiency in macrophages has an important consequences for the maintenance of organism homeostasis as well as for the resolution of pathological conditions. The latter was evidenced in a model of experimental autoimmune encephalomyelitis (EAE). The LysM^Cre/+^Hmox1^fl/fl^ mice developed more severe symptoms of EAE, what was correlated with exacerbated autoimmune T-cell response against myelin [117].

The connection of altered HO-1 expression in monocyte/macrophage lineage and autoimmune diseases are also supported by clinical observations. CD14^+^ monocytes from patients with autoimmunological systemic lupus erythematosus (SLE) have decreased HO-1 expression both at mRNA and protein level [118].

Studies performed by our group showed that lack of HO-1 disturbs also granulocyte numbers. HO-1^−/−^ mice have more granulocytes in peripheral blood, what is connected with disordered maturation of granulocytes in the bone marrow. Myelocytes, the last dividing precursor stage in granulopoiesis, proliferate faster in HO-1^−/−^ mice. Consistently, HO-1^−/−^ myelocytes have a higher expression of C/EBPβ transcription factor, which drives the granulocyte differentiation [119].

Apart from important function in the regulation of innate immunity, HO-1 is also implicated in the regulation of adaptive immune response. First, HO-1 was shown to affect the maturation of dendritic cells (DC). HO-1 induction reduced the antigen presentation by DC and inhibited their pro-inflammatory function [120]. HO-1 in DC conserves IL-10 expression that is connected with anti-inflammatory DC phenotype [121]. The mechanism of anti-inflammatory role of HO-1 in DC was linked to CO-dependent reduction of TLR signaling [122].

As expected, the inhibition of DC maturation by HO-1 leads to reduced T-cell toxicity. This was shown in a model of transgenic mice that have autoreactive CD8^+^ T cells against insulin [122]. Such CD8^+^ T-cells induce diabetes after adoptive transfer only when previously immunized with DC. However, when DC were overexpressing HO-1, the CD8^+^ cells lost the ability to induce diabetes [122]. This confirms a crucial role of HO-1 in DC in regulation of the adaptive immunity.

Importantly, a lot of the studies concerning the influence of HO-1 on dendritic cells development/activity, including the recent ones, were done using CoPP (cobalt protoporphyrin IX) as an HO-1 activator. As shown already in 2008, some of the CoPP-induced effects on DC are HO-1 independent [123], so the conclusions from those experiments must be taken with caution.

Finally, HO-1 was proposed to regulate the function of suppressive T regulatory cells (Tregs). It was revealed that CD4^+^ CD25^+^ Tregs have a constant expression of HO-1 [124]. The pharmacological inhibition of HO-1 in Tregs diminished their suppressive function [125]. Nevertheless, the next studies raised doubts about HO-1 role in Tregs, as the Tregs isolated from HO-1^−/−^ mice showed normal suppressive activity [126]. It was proposed, that it is HO-1 expression in DC that is required for Treg function, rather than intrinsic HO-1 in Tregs [127].

## HO-1 and mobilization of bone marrow cells to peripheral blood

In steady-state conditions, mature blood cells are released from the bone marrow to the blood to sustain hematological homeostasis [128]. The bone marrow provides a barrier to limit egress of immature cells to peripheral blood and allows the circulation of only small numbers of hematopoietic stem cells [128]. In response to stress stimuli, such as inflammation or neutropenia, as well as in pathological conditions, such as developing tumor, hematopoiesis is accelerated and more cells are released from the bone marrow to the circulation [128] in a process called mobilization [129]. Several cytokines, such as SCF (stem cell factor), VEGF, IL-3, IL-6, GM-CSF and M-CSF regulate the mobilization of bone marrow cells, however, the most important are G-CSF and SDF-1α [129]. G-CSF increases the proliferation of the bone marrow myeloid progenitors as well as differentiation toward granulocytic lineage [130].

The influence of the HO-1 deficiency on the effect of G-CSF-induced mobilization is not clear. We observed that wild-type mice mobilize granulocytes better than HO-1^−/−^ mice in response to G-CSF treatment [119], whereas the study by Ratajczak’s laboratory shows a rather opposite effect [131]. The possible reason for this discrepancy might be the genetic background—C57BL/6 × FVB [119] vs. 129 Sv × BALB/c [131] or model differences—the KO mice which we are using have elevated basal granulocyte numbers compared to wild type littermates [48] and such effect was not observed in the study by Ratajczak’s group [131].

Two papers from Ratajczak’s laboratory show the modulation of mobilizing factors effect by pharmacological modulation of HO-1 activity. Injection of HO-1 inhibitor (SnPP) together with standard mobilizing factors, G-CSF or plerixafor, potentiated their mobilizing effect [131]. Oppositely, treatment of mice with HO-1 inducer (CoPP) lead to the decreased mobilization of neutrophils from the bone marrow to lungs in response to LPS [132].

Another study from the same group showed that ex vivo incubation of BM-MNCs with HO-1 activity inhibitor, SnPP, resulted in their increased homing to BM after transplantation to the irradiated mice [133]. However, the experiments with protoporphyrins must be evaluated with caution, as CoPP given alone increases the expression of endogenous G-CSF and induces mobilization of granulocytes and HSP [134]. Using HO-1 deficient mice we showed that this effect is independent of HO-1 [134].

## HO-1 independent effects of protoporphyrins

CoPP is a known HO-1 inducer, used in many in vitro and in vivo studies and usually the effects of CoPP have been attributed to HO-1 activity. CoPP activates HO-1 expression by destabilization of Bach1 repressor and stabilization of the Nrf2 transcription factor [135]. Although CoPP is not found in normal conditions in the organism, it may be formed in vivo after CoCl_2_ injection [136]. Metalloporphyrins in which iron is replaced by other metals, are able to induce HO-1 expression at mRNA and protein level, just as heme does [137, 138]. However, at the same time non-heme porphyrins are the competitive inhibitors of HO-1 [137–139]. The increase in HO-1 expression induced by CoPP prevails over the transient inhibitory effect of CoPP on HO-1 activity [137]. Oppositely, SnPP inhibits HO-1 activity more strongly than CoPP and at the same time less potently upregulates HO-1 expression [137]. Overall, concerning the final effect, CoPP is used as HO-1 inducer and SnPP as HO-1 inhibitor, but one has to remember that both can exert similar activities. The level of HO-1 induction by metalloporphyrins as well as its duration depend on the metal [137, 139]. CoPP is a rapid inducer of *Hmox1* mRNA, but the maximal induction of HO-1 activity occurs at the later time than after stimulation with heme and lasts longer [139, 140].

The possibility that porphyrins may have several activities that are independent of their HO-1 modulatory function was already suggested by us and others. Our group showed that two HO-1 inhibitors (SnPP and ZnPP) have opposite effects on nitric oxide production by iNOS, after the stimulation of cells with IL-1β or LPS [141]. Blumenthal and coworkers reported that both CoPP and SnPP can directly inhibit caspase-3 and -8 activity, independently of HO-1 [138]. Both of these caspases were shown to play a role in hematopoiesis [142]. Inhibition of caspase-3 and -8 decreases human neutrophil apoptosis [143].

Different metalloporphyrins may also have different effects on the bone marrow cells—zinc porphyrins (ZnPP and ZnMP) where shown to inhibit hematopoiesis in vitro, whereas other HO-1 inbibitors—tin porphyrins had no effect [144].

Two other studies indicate the HO-1-independent modulation of immune reaction by CoPP. One of them shows that CoPP inhibited LPS-stimulated activation of inducible nitric oxide synthase (iNOS) in RAW264.7 macrophages by blocking JNK phosphorylation [145]. Silencing of HO-1 with siRNA did not affect iNOS inhibition by CoPP [145]. Similarly, cyclooxygenase-2 (COX-2) upregulation by CoPP in microglia was proved to be HO-1-independent by the use of HO-1 siRNA [146].

Those findings indicate that the conclusions about the HO-1 influence on a given process based on protoporphyrin treatment need to be confirmed using genetic models.

## Conclusions

HO-1 plays an important role in the maintenance of the hematopoiesis, ensures the proper function of the HSC niche and regulates the differentiation of progenitors to mature blood cells. HO-1 affects the immune system by directly influencing the immune cells, but also by influencing the immunomodulatory function of other cell types involved in immune response, such as endothelial or mesenchymal stromal cells.
